# Supplementary material for: Visible-Light-Active N-Doped TiO2 Photocatalysts: Synthesis from TiOSO4, Characterization, and Enhancement of Stability Via Surface Modification
Source: Nanomaterials (Basel). 2022 Nov 23;12(23):4146. doi: 10.3390/nano12234146 (PMC9739126; doi:10.3390/nano12234146)
Supplement: Supplementary file 1 [file nanomaterials-12-04146-s001.zip › nanomaterials-2030211-supplementary.pdf]

# Visible-Light-Active N-doped $TiO_2$ Photocatalysts: Synthesis from $TiOSO_4$, Characterization, and Enhancement of Stability via Surface Modification

Nikita Kovalevskiy[a], Dmitry Svintsitskiy[a], Svetlana Cherepanova[a], Stanislav Yakushkin[a], Oleg Martyanov[a], Svetlana Selishcheva[a], Evgeny Gribov[a], Denis Kozlov[a], Dmitry Selishchev[a,*]

[a]Boreskov Institute of Catalysis, Novosibirsk 630090, Russian Federation

## Supplementary Materials

### 1. Photocatalytic tests

Acetone was selected as a test organic substrate for the photocatalytic experiments due to it does not cause the deactivation of photocatalyst and is completely oxidized to $CO_2$ and water without gaseous intermediates that allows valid evaluation of photocatalytic activity and light utilization efficiency using the rate of $CO_2$ formation due to $CO_2$ has much higher attenuation coefficient than acetone and results in more accurate values. To monitor the concentrations of acetone and $CO_2$ during the experiment, the IR spectra were collected periodically every 30 s. The quantitative analysis was performed by the integration of collected IR spectra using the Beer-Lambert law as follows:

$$\int_{\omega_1}^{\omega_2} A(\omega)\,d\omega = \varepsilon \times l \times C$$

where $A(\omega) = \lg\left(\frac{I_0(\omega)}{I(\omega)}\right)$ is the absorbance, $\omega_1$ and $\omega_2$ are the limits of the corresponding absorption bands (cm$^{-1}$), $\varepsilon$ is the attenuation coefficient (μmol$^{-1}$ L cm$^{-2}$), l is the optical path length (cm), and C is the concentration of a substance in the gas phase (μmol L$^{-1}$). The regions for calculation were selected as follows: 1160–1265 cm$^{-1}$ for acetone and 2200–2400 cm$^{-1}$ for $CO_2$. The attenuation coefficients for each substance were calculated from the calibration data.

Before the evaluation of photocatalytic activity, the adsorption-desorption equilibrium of acetone on the photocatalyst was achieved until no difference in inlet and outlet acetone concentrations was observed. After that, a light source was turned on and the photocatalytic activity was evaluated. A high-power UV LED with a maximum at 371 nm and blue LED with a maximum at 450 nm were used for the photocatalyst irradiation. Figure S1a and b show the

emission spectra of the diodes that are measured using an ILT 950 spectroradiometer from International Light Technologies Inc. (USA). The total irradiance was 9.7 mW cm$^{-2}$ for UV-LED and 164 mW cm$^{-2}$ for Vis-LED.

Figure S1c shows a typical $CO_2$ concentration profile during the photocatalytic experiment and a range for evaluation of steady-state activity.

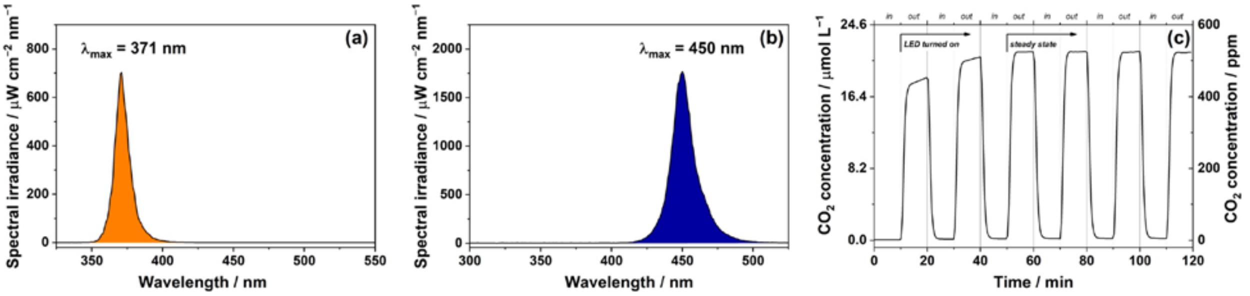


**Figure S1**. Emission spectra of the diodes used for irradiation of photocatalysts (**a,b**) and a typical $CO_2$ concentration profile during the photocatalytic experiment (**c**).

## 2. Analysis of XRD patterns

Consideration of only the size effect during the analysis of XRD patterns does not give accurate fitting of the experimental patterns of synthesized samples (Figure S2). Therefore, the estimation of crystallite size was performed considering the effect of strains according to the Le Bail fitting method. Using this approach, a good agreement between fitted and experimental patters was observed.

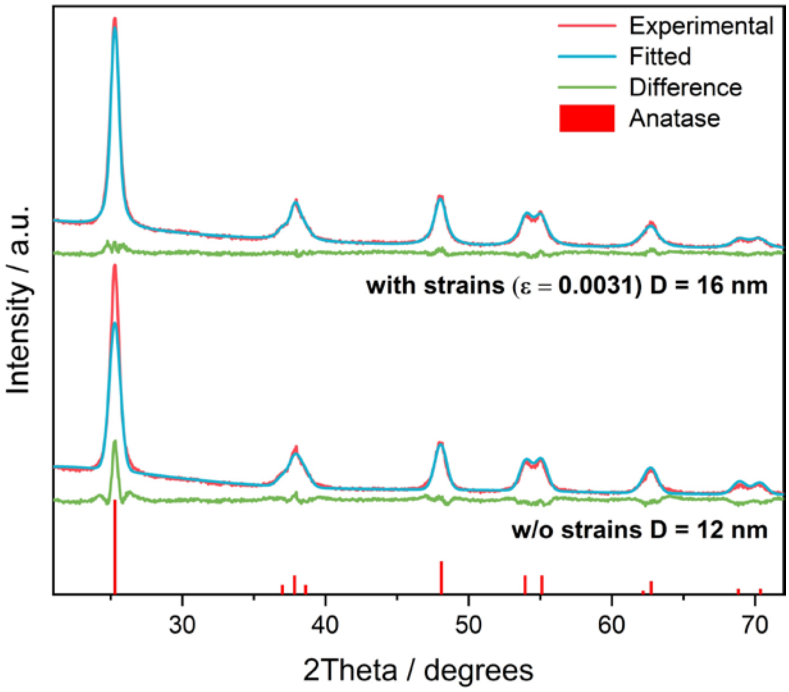


**Figure S2**. Experimental and fitted XRD patterns of the $TiO_2$-N sample synthesized at 350 °C.

The diffraction peaks of N-doped $TiO_2$ are slightly shifted compared to $TiO_2$ sample because the constants of the tetragonal lattice (a and c) and, consequently, the volume of the unit cell (V) are changed (Table S1). A greater content of nitrogen led to a higher value of strains (i.e., microdeformations) but a lower value of unit cell volume (V).

**Table S1**. Average crystallite sizes (D), strain values (ε), lattice constants (a and c), volumes of unit cell (V) for $TiO_2$ and $TiO_2$-N samples calcined at different temperatures.

| Sample | D, nm | ε | a, Å | c, Å | V, $Å^3$ |
|---|---|---|---|---|---|
| $TiO_2$ (350 °C) | 15 | 0.0020 | 3.790(1) | 9.497(2) | 136.4(1) |
| $TiO_2$-N (350 °C) | 17 | 0.0031 | 3.786(1) | 9.471(2) | 135.8(1) |
| $TiO_2$-N (500 °C) | 23 | 0.0017 | 3.787(1) | 9.498(1) | 136.2(1) |
| $TiO_2$-N (600 °C) | 25 | 0.0010 | 3.788(1) | 9.508(2) | 136.4(1) |

PDF#21-1272 ($TiO_2$, anatase, $I4_1/amd$, a=3.785Å, c=9.513Å, V=136.3$Å^3$)

### 3. Hydrogen evolution

For testing in hydrogen evolution, $TiO_2$-N sample synthesized at 350 °C was modified with noble metals. 1 wt.% of Pt, Pd, or Rh was deposited on the surface of $TiO_2$-N via chemical reduction of precursors ($H_2PtCl_6$, $PdCl_2$, $Rh(NO_3)_3$) with $NaBH_4$. Commercially available $TiO_2$ Aeroxide P25 (Evonik Ind., Germany) was used as a reference sample and was modified by the same way.

The experiments on the photocatalytic water splitting were performed in a batch rector with 100 mg of photocatalyst suspended in 200 mL of deionized water. Analysis of gas mixture in the reactor was performed using a GCh1000 gas chromatograph (Choromos Engineering, Russia) equipped with TCD by taken probes using a syringe.

$TiO_2$-N supported with Pt, Pd, or Rh (1 wt.%) can photocatalytically decompose water with formation of molecular hydrogen in the gas phase. Kinetic curves of hydrogen evolution under irradiation with UV light (20 mW $cm^{-2}$) are shown in Figure S3a. Under concerned conditions, activity of photocatalysts based on $TiO_2$-N is much higher than the activity of $TiO_2$ P25 supported with Pt (1 wt.%).

The activity of the synthesized catalysts was also evaluated in $H_2$ evolution from a methanol–water solution in a continuous-flow setup. In this setup, a maintained flow of helium passed through a saturator with water to smooth the effect of water evaporation. Thereafter flow of He went to the photoreactor with a LED light source on top of it. The outlet reaction mixture was analyzed using a GCh1000 gas chromatograph. Probes were collected automatically every 20

min to monitor the concentration of hydrogen. The $H_2$ evolution rate was calculated from the experimental values of $H_2$ concentration as follows:

$$W_{H_2} = C_{H_2} \times U \times \frac{P}{RT}$$

where $W_{H_2}$ is the rate of $H_2$ evolution (μmol min$^{-1}$), $C_{H_2}$ is the volume fraction of $H_2$ in outlet flow, U is the volume flow rate (cm$^3$/min), R is the gas constant ($8.2 \times 10^{-5}$ cm$^3$ atm μmol$^{-1}$ K$^{-1}$), T is the temperature of chromatograph calibration (298 K), P is the atmospheric pressure (accepted as 1 atm).

Under irradiation of metal-supported photocatalysts (50 mg) with 20 mW cm$^{-2}$ of UV light in the continuous-flow setup, evolution of hydrogen occurred with a high rate for long time (see Figure S3b), but in this case the activity of samples based on $TiO_2$-N was lower than the activity of reference sample (i.e., 1%Pt/$TiO_2$ P25).

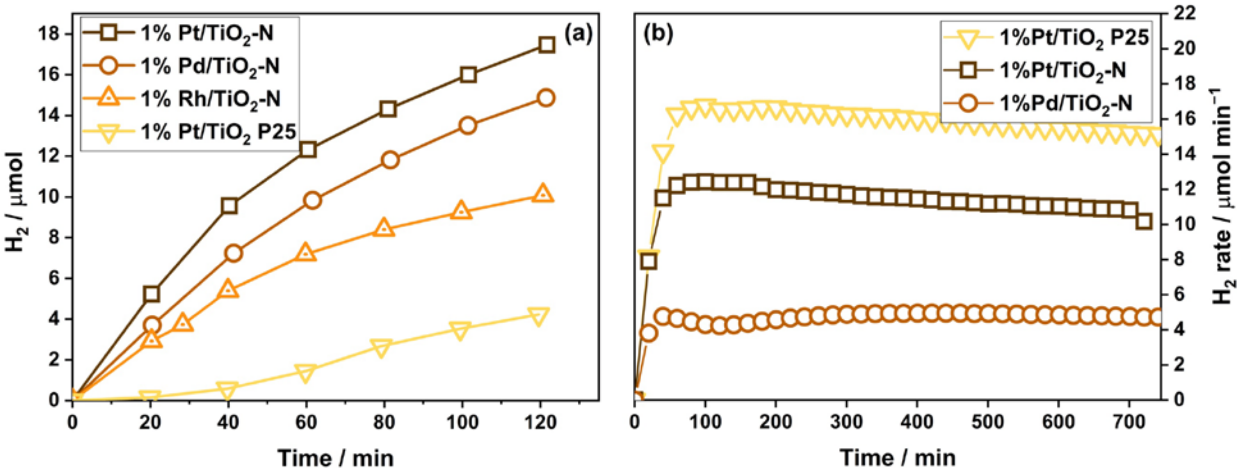


**Figure S3**. Photocatalytic evolution of hydrogen from pure water (**a**) and a methanol–water solution (**b**) over $TiO_2$-N and $TiO_2$ P25 modified with noble metals.

In both regimes mentioned above, evolution of hydrogen over $TiO_2$-N-based photocatalysts can be also achieved under blue light (450 nm, 100 mW cm$^{-2}$), but the reaction rates are much lower compared to UV.

## 4. Analysis of DRIFT spectra

All catalysts calcined at different temperatures, as well as an uncalcined sample, were studied by DRIFTS. All samples are characterized by bands at 3690 and 1640 cm$^{-1}$, which are assigned to OH-groups on the surface of $TiO_2$.

For the uncalcined sample, a broad peak at 1440 cm$^{-1}$ and a very weak peak at 1323 cm$^{-1}$ are observed (Figure S4). These peaks can be attributed to ammonia adsorbed on the surface [1]. In addition, we previously studied bands of adsorbed ammonia on anatase $TiO_2$ Hombifine N and bands of photocatalytic oxidation products. It was shown that during ammonia adsorption a broad

band at 1450 cm$^{-1}$ appears together with the band at 1327 cm$^{-1}$, which are very close to the bands observed at 1440 cm$^{-1}$ and 1323 cm$^{-1}$.

For the calcined catalysts, such bands as 1357 cm$^{-1}$ (bidentate nitrate [2]), 1387 cm$^{-1}$ (monodentate nitrite [3]), 1430 cm$^{-1}$ (monodentate nitrate [1,2,4]), 1552 cm$^{-1}$ (bidentate nitrate [1,4,5]), 2146 cm$^{-1}$ ($Ti^{4+}$–$NO^+$ [3]) can be detected (Figure S4).

The band at 1552 cm$^{-1}$ was found for all calcined samples, however, for samples at 250 and 300 °C, this peak is very weak. For an annealing temperature of 350–600 °C, the 1552 cm$^{-1}$ band has a high intensity. In addition, for samples with an annealing temperature of 350–600 °C, a weak band was found at 1357 cm$^{-1}$, which also refers to bidentate nitrate groups, as well as 1430 cm$^{-1}$, which refers to monodentate nitrate groups adsorbed on the $TiO_2$ surface. It should be noted that for samples with an annealing temperature of 250 and 300 °C, a broad band with a maximum at 1415–1430 cm$^{-1}$ was found, which, together with a weak band at 1552 cm$^{-1}$, can mean both $NO_x$ and $NH_4^+$ adsorbed on the surface [4].

Also, two bands were found at 1387 cm$^{-1}$ and 2146 cm$^{-1}$, for samples calcined at a temperature of 350–500 °C (Figure S4), which were correlated with surface monodentate nitrite and $Ti^{4+}$–$NO^+$, respectively, while the intensity maximum was observed for the sample calcined at 400 °C.

Based on the results obtained, it can be concluded that all samples calcined at temperatures above 350 °C no bands are observed that could be attributed to ammonia adsorbed on the surface. In addition, at an annealing temperature above 500 °C, all the observed bands can only be attributed to completely oxidized nitrogen. On the contrary, at a temperature of 350–500 °C, not only bands are observed that are correlated with surface nitrate groups, but also incompletely oxidized forms of nitrogen, such as nitrite and $NO^+$.

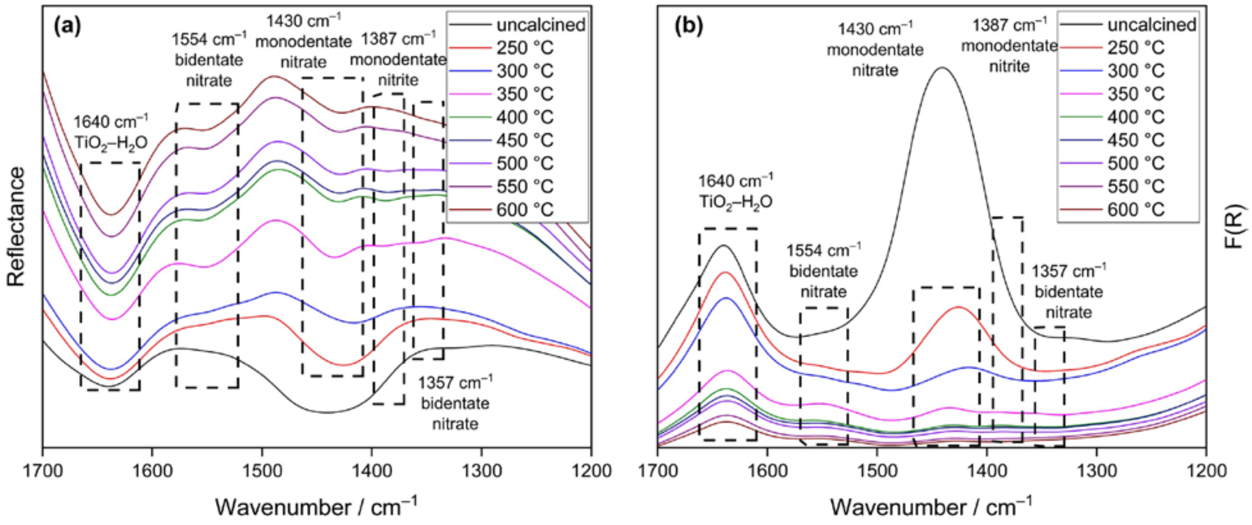


**Figure S4**. DRIFT spectra of $TiO_2$-N samples calcined at different temperatures in reflectance (**a**) and Kubelka-Munk (**b**) modes.

It is important to note that the described states were not detected on the surface of $TiO_2$-N during XPS analysis due to the treatment in vacuum in combination with long-term exposure to X-ray radiation.

## 5. Comparison of photocatalytic activity

Table S2 shows visible-light activity of $TiO_2$-N photocatalysts described in the previously published papers for comparison. A comparison of activity in the degradation of pollutants is made as a ratio to the activity of commercially available Aeroxide $TiO_2$ P25 photocatalyst used as a benchmark.

**Table S2.** Visible-light activity of $TiO_2$-N toward Aeroxide P25

| Synthesis | Pollutant | Ratio |
| --- | --- | --- |
| Ultrasound-assisted impregnation of P25 with urea [6] | Dibenzothiophene | 6 times higher |
| Grinding P25 with urea [7] | Ethylbenzene | 1.6 times higher |
| Hydrolysis of $Ti(OBu)_4$ with ammonia [8] | Phenol | 4 times higher |
| Hydrolysis of $Ti(i\text{-}OPr)_4$ with 2-methoxyethylamine [9] | Methylcyclohexane | 10 times higher |
| Hydrolysis of $Ti(OBu)_4$ with hydrazine [10] | Methyl orange | 2 times higher |
| Hydrolysis of $Ti(OBu)_4$ with ammonia [11] | Phenol | 12 times higher |
| Hydrolysis of $Ti(OBu)_4$ with ethylenediamine [12] | Methyl orange | 26 times higher |
| | Methylene blue | 10 times higher |
| Annealing $TiO_2$ nanotubes in $NH_3$ flow [13] | Methylene blue | 4 times higher |

| | | |
|---|---|---|
| Annealing P25 impregnated with tri-thiocyanuric acid [14] | $NO_x$ | 3 times higher |
| Hydrolysis of $Ti(OBu)_4$ with $NH_4F$ [15] | Methylene blue | 4 times higher |
| $Ti(OBu)_4$ + $N_2$ with plasma [16] | Toluene | 5 times higher |
| Hydrolysis of $Ti(i\text{-}OPr)_4$ with 1-ethyl-3-methylimidazolium chloride [17] | Hydrogen production | 22 times higher |
| Hydrolysis of $TiOSO_4$ with ammonia [18] | Benzyl alcohol | 10 times higher |
| Hydrolysis of $TiOSO_4$ with ammonia [19] | 4-nitrophenol | 6 times higher |
| Hydrolysis of $TiOSO_4$ with urea [20] | Methyl orange | 3 times higher |
| | Methylene blue | 3 times higher |
| This study | Acetone | 40 times higher |

## 6. Effect of Fe on the activity of $TiO_2$-N under visible light

$TiO_2$-N sample synthesized at 350 °C was modified using Fe (see the main text of article). The content of Fe was varied from 0.01 wt.% to 0.5 wt.%. Further, the prepared samples were investigated in a test rection of acetone oxidation under visible light similarly to the conditions described in the main text of article).

The effect of Fe content on the photocatalytic activity of Fe/$TiO_2$-N under visible light is shown in Figure S5. This figure shows that an optimum content of Fe is to be in a range of 0.1–0.3 wt.%.

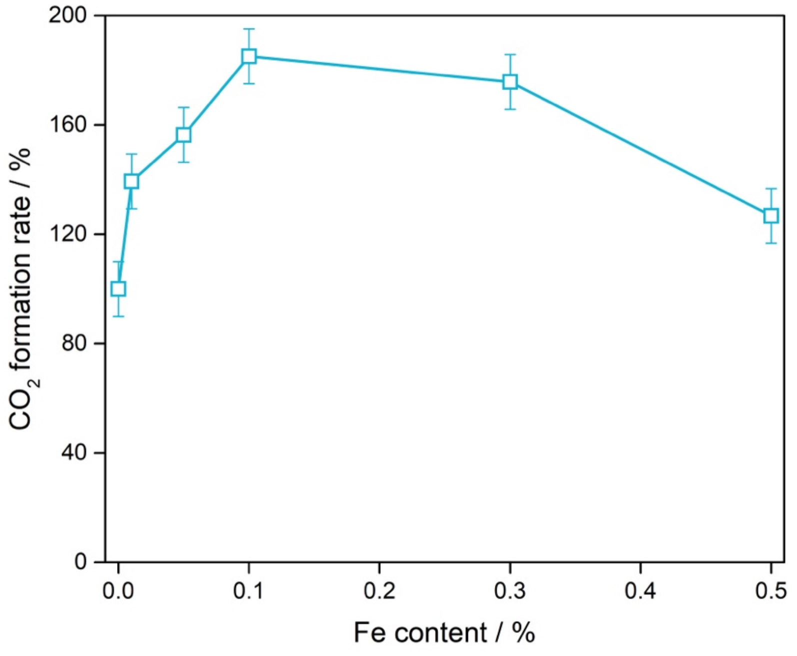


**Figure S5**. Effect of Fe content on the visible-light activity of Fe/$TiO_2$-N

The synthesized Fe/$TiO_2$-N sample with 0.3 wt.% of Fe was investigated by XPS. The results are shown in Figure S6. The peaks with an energy of 709.8 (Fe2$p_{3/2}$), 715.1 (Fe2$p_{3/2}$ satellite), and 722.4 eV (Fe2$p_{1/2}$) were detected. Fe2$p_{3/2}$ satellite with an energy of 715.1 eV allows us to establish that the observed peak can be attributed to $Fe^{2+}$ state [21].

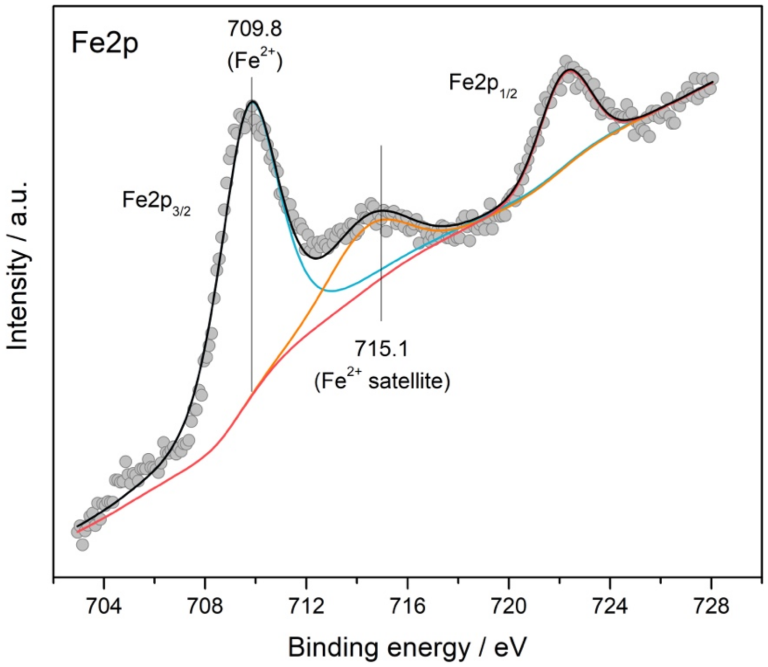


**Figure S6**. Photoelectron Fe2p spectral region for 0.3% Fe/$TiO_2$-N sample.

## References


[1] S.M. Jung, P. Grange, The investigation of mechanism of SCR reaction on a TiO2-SO42− catalyst by DRIFTS, Appl Catal B. 27 (2000) L11–L16. https://doi.org/10.1016/S0926-3373(00)00145-4.

[2] P.A. Kolinko, D. v. Kozlov, Products distribution during the gas phase photocatalytic oxidation of ammonia over the various titania based photocatalysts, Appl Catal B. 90 (2009) 126–131. https://doi.org/10.1016/J.APCATB.2009.03.001.

[3] X. Tan, G. Cheng, X. Song, X. Chen, W. Dai, X. Fu, The promoting effect of visible light on the CO + NO reaction over the Pd/N–TiO2 catalyst, Catal Sci Technol. 9 (2019) 3637–3646. https://doi.org/10.1039/C9CY00466A.

[4] G. Ramis, G. Busca, V. Lorenzelli, P. Forzatti, Fourier transform infrared study of the adsorption and coadsorption of nitric oxide, nitrogen dioxide and ammonia on TiO2 anatase, Appl Catal. 64 (1990) 243–257. https://doi.org/10.1016/S0166-9834(00)81564-X.

[5] R. Jin, Y. Liu, Y. Wang, W. Cen, Z. Wu, H. Wang, X. Weng, The role of cerium in the improved SO2 tolerance for NO reduction with NH3 over Mn-Ce/TiO2 catalyst at low temperature, Appl Catal B. 148–149 (2014) 582–588. https://doi.org/10.1016/J.APCATB.2013.09.016.

[6] K. Kalantari, M. Kalbasi, M. Sohrabi, S.J. Royaee, Synthesis and characterization of N-doped TiO2 nanoparticles and their application in photocatalytic oxidation of dibenzothiophene under visible light, Ceram Int. 42 (2016) 14834–14842. https://doi.org/10.1016/j.ceramint.2016.06.117.

[7] M. Kamaei, H. Rashedi, S.M.M. Dastgheib, S. Tasharrofi, Comparing Photocatalytic Degradation of Gaseous Ethylbenzene Using N-doped and Pure TiO2 Nano-Catalysts Coated on Glass Beads under Both UV and Visible Light Irradiation, Catalysts 2018, Vol. 8, Page 466. 8 (2018) 466. https://doi.org/10.3390/CATAL8100466.

[8] W. Zhengpeng, G. Wenqi, H. Xiaoting, C. Weimin, J. Juhui, Z. Baoxue, Preparation, characterization and visible light photocatalytic activity of nitrogen-doped $TiO_2$, Journal of Wuhan University of Technology-Mater. Sci. Ed. 21 (2006) 71–77. https://doi.org/10.1007/BF02841209.

[9] C. Belver, R. Bellod, S.J. Stewart, F.G. Requejo, M. Fernández-García, Nitrogen-containing TiO2 photocatalysts: Part 2. Photocatalytic behavior under sunlight excitation, Appl Catal B. 65 (2006) 309–314. https://doi.org/10.1016/J.APCATB.2006.02.016.

[10] X. Li, G. Zhang, X. Wang, W. Liu, K. Yu, C. Liang, Facile synthesis of nitrogen-doped titanium dioxide with enhanced photocatalytic properties, Mater Res Express. 6 (2019) 115019. https://doi.org/10.1088/2053-1591/AB44F2.

[11] Z. Wang, W. Cai, X. Hong, X. Zhao, F. Xu, C. Cai, Photocatalytic degradation of phenol in aqueous nitrogen-doped TiO2 suspensions with various light sources, Appl Catal B. 57 (2005) 223–231. https://doi.org/10.1016/J.APCATB.2004.11.008.

[12] G. Yang, Z. Jiang, H. Shi, T. Xiao, Z. Yan, Preparation of highly visible-light active N-doped TiO2 photocatalyst, J Mater Chem. 20 (2010) 5301–5309. https://doi.org/10.1039/C0JM00376J.

[13] Y. Wang, J. Zhang, Z. Jin, Z. Wu, S. Zhang, Visible light photocatalytic decoloration of methylene blue on novel N-doped TiO2, Chinese Science Bulletin 2007 52:15. 52 (2007) 2157–2160. https://doi.org/10.1007/S11434-007-0306-X.

[14] T.T. Khan, G.A.K.M. Rafiqul Bari, H.J. Kang, T.G. Lee, J.W. Park, H.J. Hwang, S.M. Hossain, J.S. Mun, N. Suzuki, A. Fujishima, J.H. Kim, H.K. Shon, Y.S. Jun, Synthesis of N-Doped TiO2 for Efficient Photocatalytic Degradation of Atmospheric NOx, Catalysts 2021, Vol. 11, Page 109. 11 (2021) 109. https://doi.org/10.3390/CATAL11010109.

[15] H.Y. Ai, J.W. Shi, R.X. Duan, J.W. Chen, H.J. Cui, M.L. Fu, Sol-gel to prepare nitrogen doped TiO2 nanocrystals with exposed {001} facets and high visible-light photocatalytic performance, International Journal of Photoenergy. 2014 (2014). https://doi.org/10.1155/2014/724910.

[16] C. Chen, H. Bai, S.M. Chang, C. Chang, W. Den, Preparation of N-doped TiO2 photocatalyst by atmospheric pressure plasma process for VOCs decomposition under UV and visible light sources, Journal of Nanoparticle Research 2006 9:3. 9 (2006) 365–375. https://doi.org/10.1007/S11051-006-9141-2.

[17] S.H. Liu, W.T. Tang, W.X. Lin, Self-assembled ionic liquid synthesis of nitrogen-doped mesoporous TiO2 for visible-light-responsive hydrogen production, Int J Hydrogen Energy. 42 (2017) 24006–24013. https://doi.org/10.1016/J.IJHYDENE.2017.08.009.

[18] M. Japa, D. Tantraviwat, W. Phasayavan, A. Nattestad, J. Chen, B. Inceesungvorn, Simple preparation of nitrogen-doped TiO2 and its performance in selective oxidation of benzyl alcohol and benzylamine under visible light, Colloids Surf A Physicochem Eng Asp. 610 (2021) 125743. https://doi.org/10.1016/J.COLSURFA.2020.125743.

[19] M. Bellardita, M. Addamo, A. di Paola, L. Palmisano, A.M. Venezia, Preparation of N-doped TiO2: characterization and photocatalytic performance under UV and visible light, Physical Chemistry Chemical Physics. 11 (2009) 4084–4093. https://doi.org/10.1039/B816708G.

[20] K.M. Parida, B. Naik, Synthesis of mesoporous TiO2 − xNx spheres by template free homogeneous co-precipitation method and their photo-catalytic activity under visible light

illumination, J Colloid Interface Sci. 333 (2009) 269–276. https://doi.org/10.1016/J.JCIS.2009.02.017.

[21] T. Yamashita, P. Hayes, Analysis of XPS spectra of Fe2+ and Fe3+ ions in oxide materials, Appl Surf Sci. 254 (2008) 2441–2449. https://doi.org/10.1016/J.APSUSC.2007.09.063.
